# Phosphodiesterase-5 inhibitors and the heart: compound cardioprotection?

**DOI:** 10.1136/heartjnl-2017-312865

**Published:** 2018-03-08

**Authors:** David Charles Hutchings, Simon George Anderson, Jessica L Caldwell, Andrew W Trafford

**Affiliations:** Unit of Cardiac Physiology, Division of Cardiovascular Sciences, School of Medical Sciences, Faculty of Biology, Medicine and Health, The University of Manchester, Manchester Academic Health Science Centre, Manchester, UK

**Keywords:** myocardial disease basic science, cardiac risk factors and prevention, cardiac arrhythmias and resuscitation science, coronary artery disease, heart failure

## Abstract

Novel cardioprotective agents are needed in both heart failure (HF) and myocardial infarction. Increasing evidence from cellular studies and animal models indicate protective effects of phosphodiesterase-5 (PDE5) inhibitors, drugs usually reserved as treatments of erectile dysfunction and pulmonary arterial hypertension. PDE5 inhibitors have been shown to improve contractile function in systolic HF, regress left ventricular hypertrophy, reduce myocardial infarct size and suppress ischaemia-induced ventricular arrhythmias. Underpinning these actions are complex but increasingly understood cellular mechanisms involving the cyclic GMP activation of protein kinase-G in both cardiac myocytes and the vasculature. In clinical trials, PDE5 inhibitors improve symptoms and ventricular function in systolic HF, and accumulating epidemiological data indicate a reduction in cardiovascular events and mortality in PDE5 inhibitor users at high cardiovascular risk. Here, we focus on the translation of underpinning basic science to clinical studies and report that PDE5 inhibitors act through a number of cardioprotective mechanisms, including a direct myocardial action independent of the vasculature. We conclude that future clinical trials should be designed with these mechanisms in mind to identify patient subsets that derive greatest treatment benefit from these novel cardioprotective agents.

## Introduction

**Figure 1:**
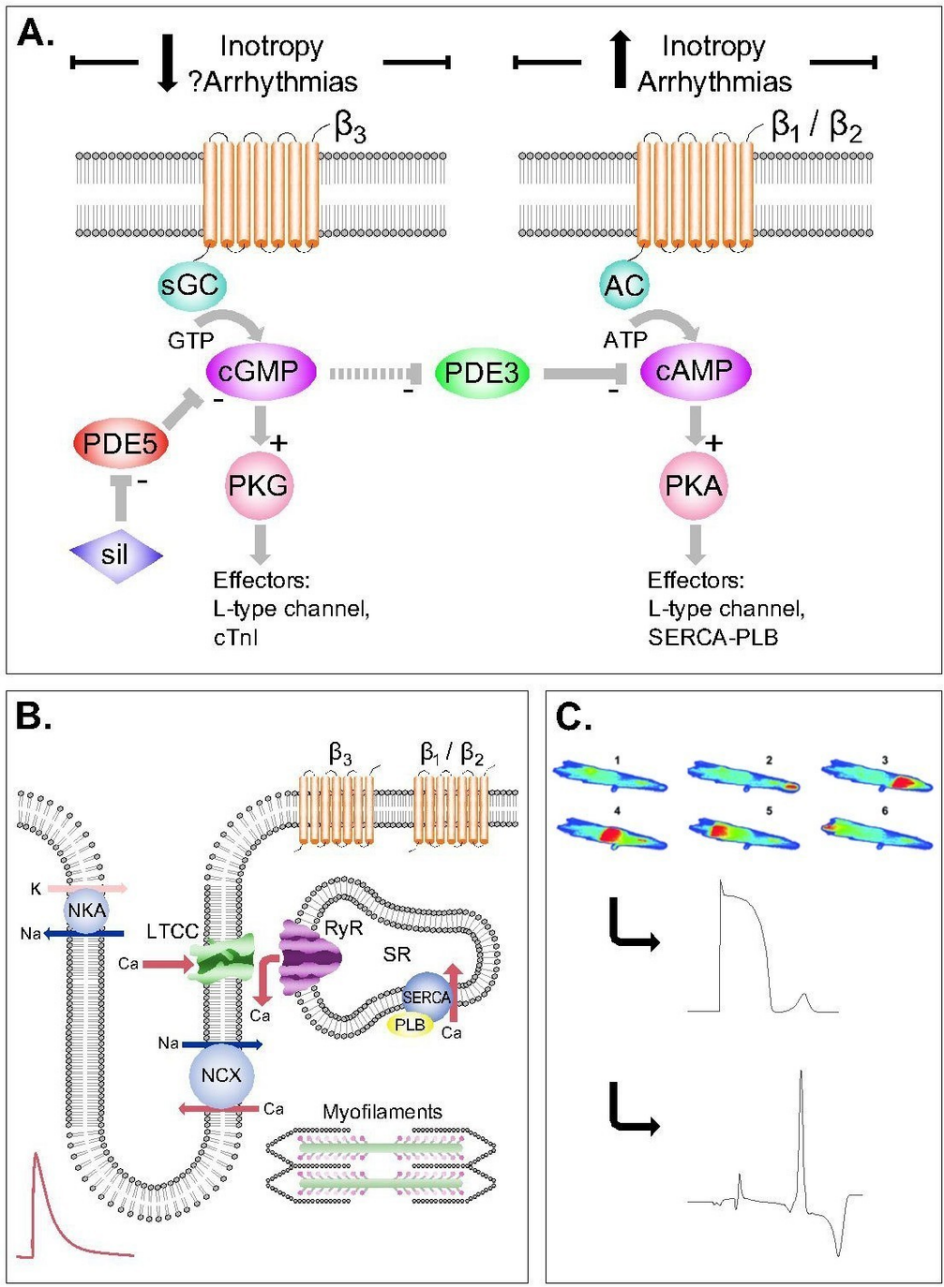
Cyclic nucleotide signalling in cardiac myocytes and its relation to contraction and arrhythmias. (A) Competing effects of cyclic GMP (cGMP) and cyclic AMP (cAMP) within the cardiac myocyte. Sildenafil (left side) inhibits phosphodiesterase-5 (PDE5) leading to the accumulation of cGMP within the cell. cGMP activates protein kinase-G (PKG) resulting in phosphorylation of the L-type Ca^2+^ channel and cardiac troponin I (cTnI). These effects are negatively inotropic. cAMP (right), for example, following stimulation of β_1_-adrenoceptors and β_2_-adrenoceptors by epinephrine, activates PKA leading to positive inotropic and proarrhythmic actions. In addition, there is also potential for crosstalk between the two axes; cGMP can inhibit phosphodiesterase-3 (PDE3) to increase cAMP. (B) Excitation–contraction coupling in the cardiac myocyte. Opening of L-type Ca channels (LTCC) during the action potential lead to influx of Ca and stimulation of Ca release from the sarcoplasmic reticulum (SR), initiating myofilament contraction. (C) Overload of the SR, as occurs in HF and under β_1_-adrenergic and β_2_-adrenergic stimulation, leads to spontaneous Ca waves, which activate Na^+^–Ca^2+^ exchanger (NCX) channels to cause after depolarisations and triggered arrhythmias.

Progression of heart failure (HF) is driven in part by maladaptive responses of cardiac muscle to pathological stress.^1^ There is growing evidence that stimulation of a signalling pathway within cardiac myocytes, the cyclic GMP (cGMP)–protein kinase-G (PKG) axis, dampens these cardiac stress responses, and its activation can attenuate pathological hypertrophy, protect against ischaemic injury and enhance cell survival.^2^ One means of potentiating cGMP–PKG is by inhibiting the degradation of cytosolic cGMP by phosphodiesterase-5 (PDE5, figure 1). PDE5 inhibitors (PDE5is) such as sildenafil (Viagra), tadalafil (Cialis) and vardenafil (Levitra) have proven efficacy in treating erectile dysfunction and pulmonary arterial hypertension (PAH).^3^ Underlying their utility in both conditions is a well-defined action on the vasculature through enhancement of cGMP derived from nitric oxide (NO), leading to relaxation of vascular smooth muscle and vasodilatation. With discovery that PDE5 is also expressed in cardiac muscle and upregulated in hypertrophy and failure,^4^ attention has shifted towards direct myocardial effects of PDE5is.

PDE5is have proven safety profiles with low incidence of adverse cardiovascular events.^5^ We recently reported that PDE5i use in patients with type 2 diabetes (T2DM) and high cardiovascular risk was associated with reduced mortality.^6^ The effect was stronger in patients with prior MI and was associated with reduced incidence of new MI, raising the possibility that PDE5is could prevent both complications post-MI and future cardiovascular events. Subsequent similar findings were observed in a post-MI cohort showing PDE5i use was accompanied with reduced mortality and HF hospitalisation.^7^ Potential for confounding in these observational studies is high, however, and data were not collected on partner status or activity levels, both of which link to greater life expectancy. Nevertheless, growing evidence from animal models support a cardioprotective action of PDE5is, including improving contractile function in HF with reduced ejection fraction (HFrEF),^8 9^ regressing left ventricular hypertrophy (LVH),^4^ reducing infarct size in myocardial infarction (MI)^10 11^ and suppressing ventricular arrhythmias (VAs).^12^

PDE5is have consistently improved patient exercise capacity and ventricular function in trials of HFrEF^13–16^; yet, outcomes in HF with preserved ejection fraction (HFpEF) have been mixed with the largest study to date failing to demonstrate clinical benefit.^17 18^ Two factors may underlie these discrepant findings. First, PDE5is exerted an effect on left ventricular (LV) remodelling in HFrEF which was not observed in HFpEF, and second, PDE5is acutely reduce contractile function at both cellular and in vivo levels, which may further limit their use in HFpEF.^19^ Thus, it is likely that PDE5is have differing efficacy according to HF subtype (HFrEF vs HFpEF).

Although there is additional interest in PDE5is in treatment of congenital heart disease, chemotherapy cardiotoxicity and hypertension, here we outline evidence behind PDE5i cardioprotection in HFrEF, HFpEF and MI. In particular, we highlight the direct myocardial actions of these novel cardioprotective agents, with a focus on translation of cellular mechanisms to clinical actions.

## Basic myocardial actions of PDE5is

To understand the effects of PDE5is on cardiac function, we must first address the processes linking excitation of the surface membrane (the action potential) to mechanical contraction of the cardiac myocyte, termed ‘excitation–contraction coupling’ (figure 1B).^20^ Briefly, depolarisation of the cell membrane during the action potential opens voltage-gated Ca channels, causing the influx of a small quantity of Ca^2+^ into the cell (*I*_Ca-L_). This triggers a larger release of Ca from its intracellular store, the sarcoplasmic reticulum (SR), giving rise to an increase in cytosolic Ca termed the ‘Ca transient’, which activates myofilament proteins and elicits contraction. Ca transient amplitude is a major determinant of cardiac inotropy and can be modified by (1) changing the size of triggering *I*_Ca-L_ or (2) changing SR Ca content. The latter is determined largely by rate of Ca uptake via the sarcoplasmic-endoplasmic reticulum Ca ATPase pump (SERCA). While cellular Ca is tightly controlled in healthy myocytes via elegant feedback mechanisms,^20^ in disease (eg, HF) the SR can become overloaded, giving rise to spontaneous releases of Ca and the formation of Ca ‘waves’.^21^ During waves, Ca is removed by the electrogenic Na–Ca exchanger channel, generating an inward electrical current resulting in delayed after depolarisations and triggered arrhythmias (figure 1C).

## PDE5 and the GC-PKG signalling pathway

**Figure 2:**
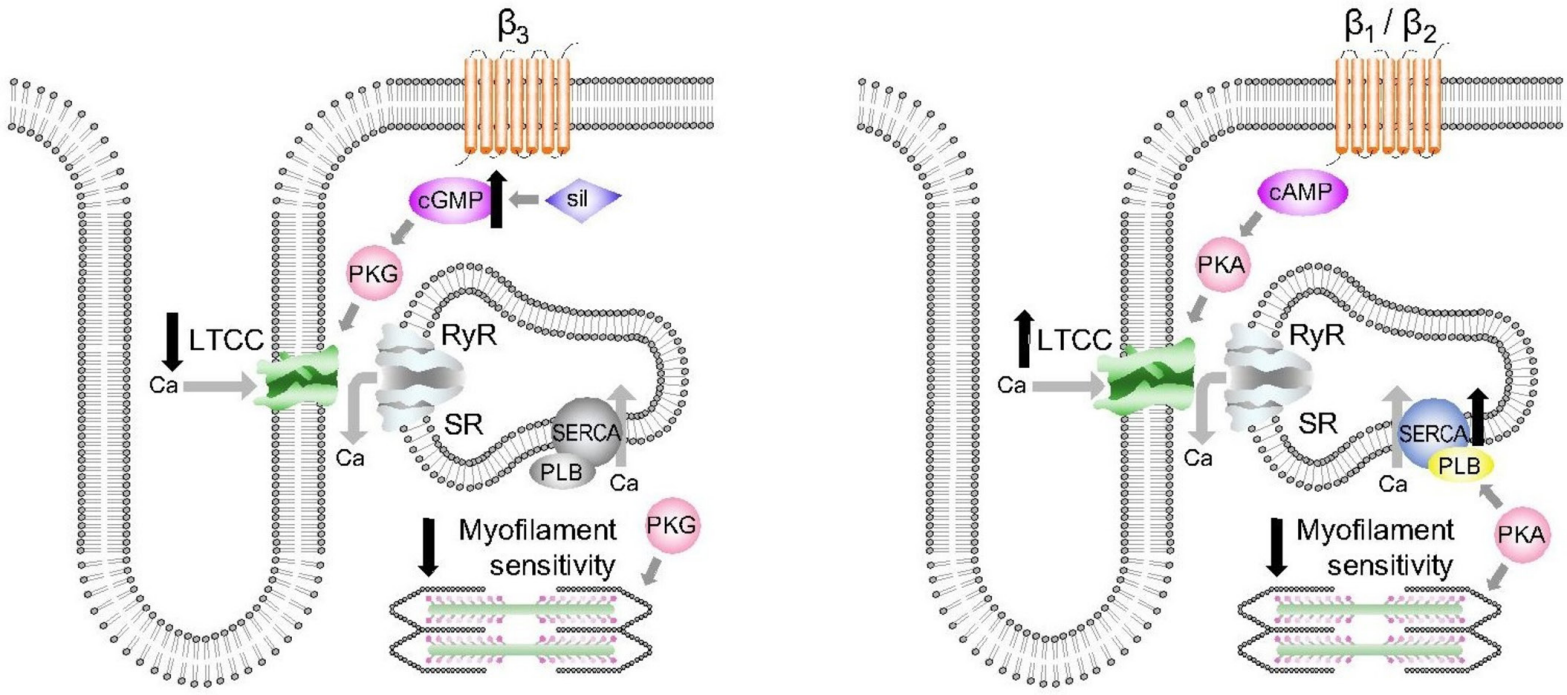
Competing effects of the cyclic GMP–protein kinase G (PKG) and cyclic AMP–protein kinase A (PKA) axes in the heart. Left panel: sildenafil increases cGMP and activates PKG, leading to phosphorylation of (1) L-type Ca channel (LTCC) to reduce Ca influx and (2) cardiac troponin I to reduce myofilament Ca sensitivity. Both effects are negatively inotropic. Right panel: β_1_-adrenergic and β_2_-adrenergic stimulation increases cAMP to activate PKA, leading to phosphorylation of (1) L-type Ca channel, increasing Ca influx and (2) phospholamban (PLB) to activate sarcoplasmic-endoplasmic reticulum Ca ATPase pump (SERCA) and increase Ca uptake into the sarcoplasmic reticulum (SR). These actions lead to a greater Ca content within the SR, increased force of contraction and greater propensity to arrhythmias.

cGMP is an intracellular second messenger generated by guanyl cyclase in response to NO stimulation (figure 1A). cGMP activates PKG which has been shown to prevent progression of pathological hypertrophy and contractile dysfunction, suppresses β-adrenergic responses and modifies signalling pathways to increase cell survival.^2^ PDE5 deactivates cGMP and therefore controls its levels, and thus PDE5is potentiate the cGMP–PKG axis.^4^ PDE5is have negative inotropic effects and oppose β-adrenergic stimulation (figure 2), by reduced myofilament Ca sensitivity via PKG-phosphorylation of cardiac troponin I,^22^ blunting β-adrenergic signalling, and reducing *I*_Ca-L_.^23^ Effects of PDE5is in whole-heart and in vivo experiments are less consistent, possibly due to PDE5i vasodilatation and consequent sympathetic activation via baroreflex activity.^24^ While some studies found no overall effect of PDE5is on contractility,^25^ a clinical trial of sildenafil in HFpEF showed an overall decrease.^19^

Cyclic-AMP (cAMP) has broadly opposing effects to cGMP. It is generated in response to epinephrine and elicits inotropic and hypertrophic responses by activating protein kinase A (PKA). As such, cAMP increases *I*_Ca-L_ and SR Ca content via increased SERCA function. These effects increase Ca transient amplitude, and, when SR content reaches threshold, cause Ca waves leading to triggered arrhythmias. Under basal conditions, the majority of cAMP deactivation is achieved by phosphodiesterase-3 (PDE3). The PDE3 binding site also has affinity for cGMP, allowing cGMP to inhibit cAMP hydrolysis,^26^ and providing a theoretical means by which cGMP can exert positive inotropic effects.

### Basic vascular effects

Vascular effects of PDE5is likely contribute to its cardioprotective actions, although full appraisal is beyond the scope of this review. Vascular tone is largely determined by availability of endothelial NO, which diffuses to adjacent smooth muscle and stimulates cGMP production leading to relaxation and vasodilatation. PDE5is increase cGMP, inducing vasodilatation. While PDE5 is particularly highly expressed in penile vasculature, it is additionally expressed in pulmonary vasculature and at lower levels in peripheral vasculature including coronary.

### Safety of PDE5is

Clinical safety and epidemiological studies have established proven safety profiles for PDE5is which are now among the top 75 dispensed drugs in the USA.^5^ The predominating safety concern relates to co-administration with nitrates resulting in profound hypotension.^3^ Cardiac effects of PDE5is were initially scrutinised over theoretical risk of arrhythmia, high proportion of users at high cardiovascular risk and case reports of VAs following PDE5i ingestion. Early concern was supported by theoretical mechanisms of proarrhythmia through (1) cGMP crosstalk with PDE3 increasing SR Ca and triggered arrhythmias, (2) block of repolarising current *I*_Kr_ prolonging repolarisation at highly supratherapeutic concentrations^27^ and (3) increased peripheral sympathetic activity.^24^ Subsequent studies of myocytes, tissue preparations and in vivo find PDE5is at therapeutic concentrations do not significantly modify *I*_Kr_ or *I*_Ks_ components of repolarisation^23^ and do not modify action potential duration or QTc.^28 29^ While PDE5is theoretically could elevate cAMP through cGMP-inhibition of PDE3, the vast majority of studies find cGMP-PKG effects predominate, with corresponding negative inotropic action.^19 22^

## PDE5is in HFrEF

**Figure 3:**
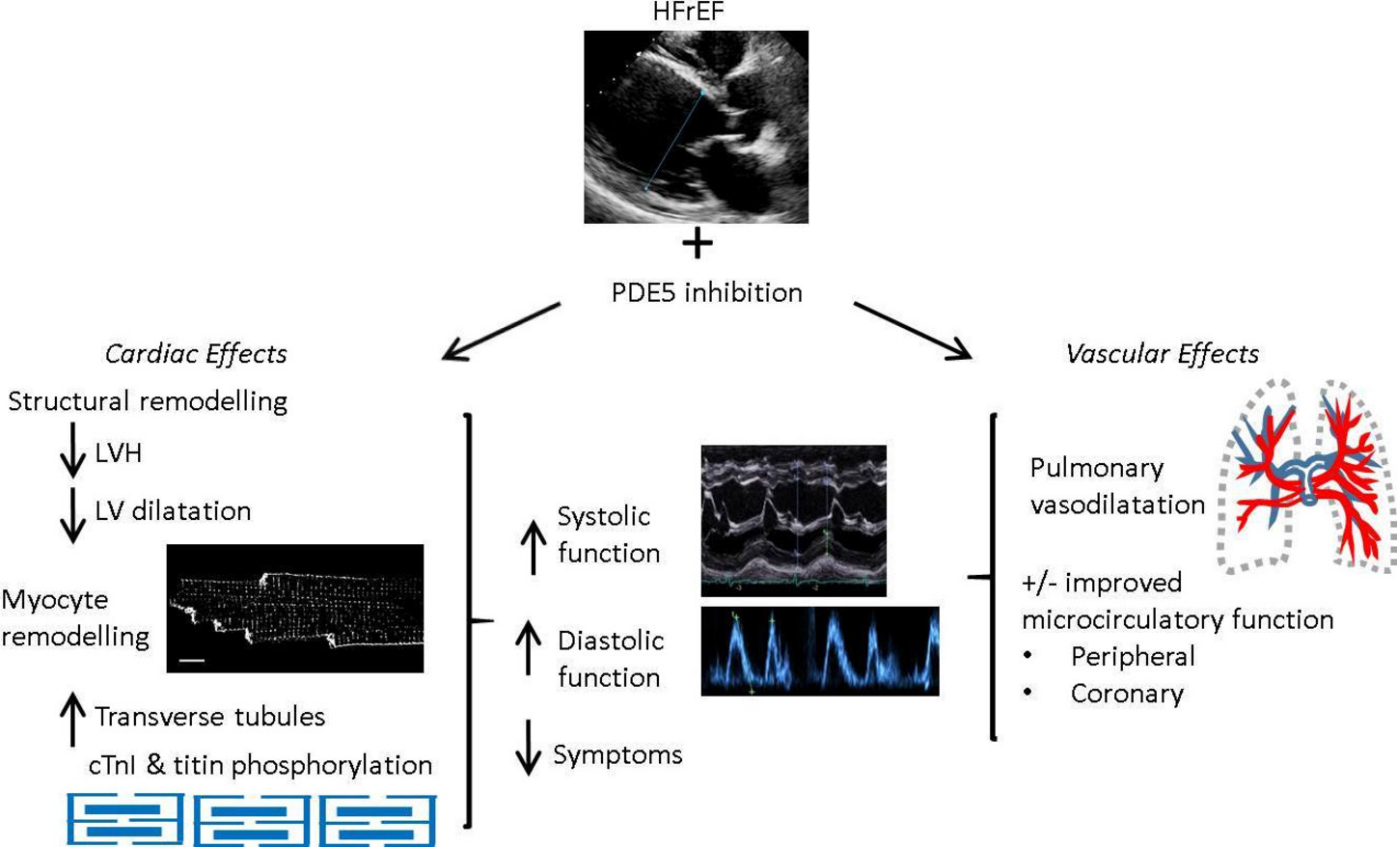
Effects of phosphodiestrase-5 (PDE5) inhibition in heart failure with reduced ejection fraction (HFrEF).

In small scale, randomised, double-blinded, placebo-controlled trials in patients with HFrEF predominantly on optimal medical therapy, treatment with PDE5is improved quality of life and exercise capacity (6 min walk distance and peak VO_2_).^13–16^ Larger randomised clinical trials of PDE5is in HFrEF are underway, including ‘SIL-HF’, a multicentre international study. Underlying these symptomatic and functional improvements are sustained increases in EF of both right and left ventricles.^14 15 30^ There appears to be a dual action underlying efficacy in HFrEF. First, an acute effect, predominantly driven by pulmonary vasodilatation, and second, a chronic effect resulting from LV remodelling independent of afterload. Underlying mechanisms are outlined below and illustrated in figure 3.

### Mechanisms underpinning efficacy of PDE5is in HFrEF

#### Pulmonary vasodilatation

HF increases peripheral and pulmonary vascular resistance. Because PDE5 is highly expressed in pulmonary vasculature and efficacious in PAH, it was suggested that greatest benefit would be attained in patients with pulmonary hypertension due to left heart disease (HFrEF-PHT). This was borne out in a pilot study by Lewis *et al* where PDE5i treatment decreased pulmonary arterial pressure both acutely and chronically, resulting in reduced pulmonary vascular resistance and increased RV EF and cardiac output.^13^

#### Cardiac remodelling

Improved contractile function, reduced chamber dilatation and reduced LVH are observed in a wide range of animal models of LV systolic dysfunction treated with PDE5is. These include mouse angiotensin II-induced HF,^9^ mouse HF induced by transverse aortic constriction (TAC)^4 31^ and mouse volume-overload HF induced by chronic mitral regurgitation.^32^ Additionally, LV structural remodelling is present at the level of the cardiac myocyte. Loss and disorganisation of t-tubules in HF is strongly associated with HF severity, results in dys-synchronous Ca transients and contractile dysfunction,^33^ and it was recently reported that sildenafil prevented these t-tubule changes in mouse systolic HF.^31^

There is strong evidence that PDE5i remodelling is due to a direct myocardial action of the drug class. While peripheral vasodilatation could contribute via reduced LV afterload, systemic blood pressure (BP) reduction by PDE5is is small in HF and may indeed be negligible making this mechanism unlikely.^30^ Furthermore, improvements in function are observed in TAC-HF models where afterload is fixed.^4^ Molecular mechanisms underlying remodelling are PKG mediated. PKG activates downstream signalling pathways controlling hypertrophy, ultimately blunting activation of a transcription factor, nuclear factor of activated T cells, which controls development of hypertrophy.^34^ Another final target of PDE5i is the RhoA-Rho-kinase pathway, again implicated in hypertrophy.^35^

Clinically, in randomised placebo-controlled trials of patients *without* significant pulmonary hypertension, PDE5i improved LVEF at 6 and 12 months treatment with little or no effect observed during acute treatment.^14 30^ Improvement in LVEF was accompanied by significant reduction in LV diameter and mass. Thus, here we conclude that PDE5is have a remodelling action on ventricular muscle that is independent of acute haemodynamic effects.

#### Diastolic function

PDE5is improve diastolic function both in animal models of systolic HF and clinical studies of HFrEF, although catheter-based assessments of diastolic dysfunction are lacking. In murine angiotension II-induced HF, sildenafil increased diastolic performance in addition to systolic, and reduced LVH.^9^ PDE5i improvements in diastolic function may be explained structurally by reducing hypertrophy, improving chamber compliance, and possible contribution of reduced fibrosis. At a cellular level, PDE5i may improve myocyte relaxation via PKG. First, via phosphorylation of titin, where, in a model of hypertensive hypertrophy, PDE5i improved diastolic compliance,^36^ and second, via reduced myofilament Ca sensitivity through PKG-phosphorylation of cardiac TnI.^22^

In a double-blinded, randomised, placebo-controlled trial of symptomatic HFrEF with echo features of diastolic dysfunction (45 patients), sildenafil improved diastolic mitral Doppler velocities and reduced ratio of early transmitral flow velocity to annular velocity (E/E′).^14^ Effects were accompanied by reversal in left atrial (LA) volume and LV mass indices. The vast majority of patients were taking β-blockers and either ACE-inhibitors or angiotensin-receptor blockers, and 42% were receiving spironolactone. Similarly, again in a randomised placebo-controlled trial, 3 month PDE5i (Udenafil) in patients with HFrEF, on optimal therapy improved E/E′ and reduced LA size in addition to improved systolic function, exercise capacity and New York Heart Association class.^15^

#### Interstitial fibrosis and inflammation

Evidence links cardiac dysfunction with inflammatory mediators in HF. A recent study demonstrated that sildenafil reduces levels of chemokine CXCL10 in T2DM and reduced CXCL10 protein and expression in cardiac myocytes.^37^ In male T2DM with LVH, Gianetta *et al* found sildenafil improved cardiac function, associated with reduction in transforming growth factor beta markers.^38^ PDE5i also reduces fibrosis in animal models of LV dysfunction accompanied by reduced inflammation.^9^ In LV dysfunction secondary to chronic mitral regurgitation in the rat, sildenafil significantly reduced perivascular fibrosis, apoptosis and hypertrophy, and transcriptional profiling revealed reduced inflammatory pathway activation.^32^

#### Coronary microcirculation

Finally, effects of PDE5is on HF coronary microcirculation, and thus O_2_ delivery, are largely unexplored but may pose a therapeutic target given that HF is accompanied by impaired coronary endothelial function, and PDE5is improve peripheral endothelial function in systolic HF patients.^39 40^

## PDE5is in HFpEF

HFpEF, defined as clinical syndrome of HF with LVEF >50%, is characterised by concentric remodelling, fibrosis and stiffness of both myocyte and extracellular matrix components of ventricular muscle. Recently proposed HFpEF paradigms implicate multiple comorbidities including T2DM, obesity, hypertension and vasculopathy, causing a systemic proinflammatory state, coronary microvascular inflammation and compromised NO availability.^41^ These favour hypertrophy and titin hyperphosphorylation, increasing myocyte stiffness.

Early promise of PDE5is was suggested by a randomised trial of 44 patients with HFpEF by Guazzi *et al*.^18^ Patients had pulmonary artery pressure (PAP) >40 mm Hg (right heart catheterisation) and RV systolic dysfunction, and PDE5i substantially improved PAP and RV function and modestly reduced PCWP suggestive of a small improvement in diastolic function. However, in Sildenafil and Diastolic Dysfunction After Acute Myocardial Infarction, a randomised trial of patients ~2 weeks post-MI with echo evidence of diastolic dysfunction (EF>45%), 9-week sildenafil treatment failed to decrease LV filling pressures although there were small reductions in N-terminal pro b-type natriuretic peptide.^42^ Unfortunately, no benefit was demonstrated in the largest randomised trial, Phosphodiesterase-5 Inhibition to Improve Clinical Status and Exercise Capacity in Heart Failure with Preserved Ejection Fraction (RELAX) trial, in patients with greatest comorbidity and widest inclusion criteria.^17^ In an ancillary study, the authors concluded PDE5i had beneficial vascular effects via improved endothelium-dependent vasodilatation,^19^ but these were offset by reductions in contractility.^19^ Reduced contractility accords with negative inotropic effects of PDE5is at the cellular level (figure 2)^22 43^ and has been demonstrated specifically in a porcine HFpEF model (aortic banding) where tadalafil reduced cell shortening and Ca transient amplitude.^44^

Several factors may reconcile differing outcomes between the Guazzi and Redfield clinical trials. First, Guazzi *et al* used patients with confirmed pulmonary hypertension (PAP >40 mm Hg) and RV systolic dysfunction showed marked improvements in right-sided pressures, improved RV function and reduced dilatation. Improvement in LV diastolic function was more modest. In contrast, Redfield *et al* included patients with multiple comorbidity, most of whom had no significant pulmonary hypertension or RV systolic dysfunction. Thus, overall positive effects of PDE5is noted by Guazzi *et al* suggest efficacy on pulmonary vasculature and RV systolic function/remodelling, with smaller effect on LV function, and in the larger study by Redfield *et al*, with no RV systolic impairment or pulmonary hypertension, no benefit was observed. Taken together, PDE5is in HFpEF have shown marginal efficacy on LV performance, with any slight improvement in diastolic function offset by small but significantly reduced contractility. Some potential could exist in a select HFpEF subset with elevated PAP and RV *systolic* dysfunction, perhaps via a similar remodelling action to that seen in LV *systolic* dysfunction, although this requires clarity in larger-scale randomised trials. It is unclear why PDE5is induce remodelling in HFrEF but not HFpEF, possibly reflective of distinct signalling pathways. It is noteworthy that cGMP production in HFpEF is substantially reduced secondary to low NO bioavailability^41^ and PDE5is may only minimally elevate plasma cGMP in HFpEF.^17^

## PDE5is in MI

**Figure 4:**
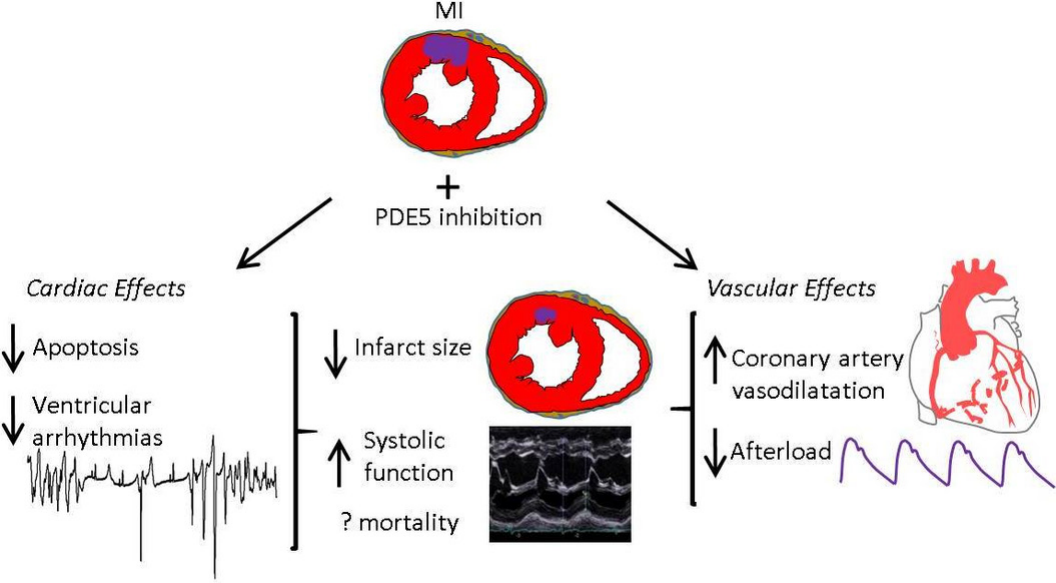
Cardioprotective effects of phosphodiesterase-5 (PDE5) inhibition in acute myocardial infarction.

**Figure 5:**
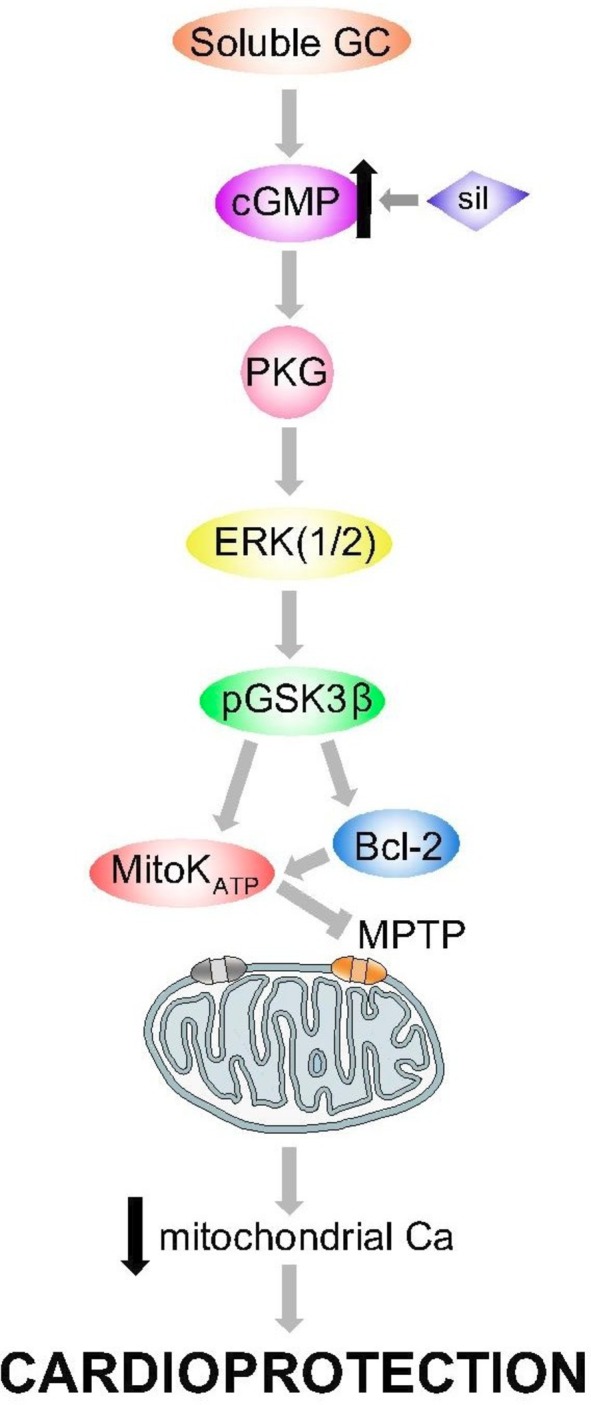
Intracellular pathway of cardioprotection in myocardial infarction as proposed by Kukreja *et al* 11.

While sildenafil was initially trialled as an antianginal, discovery of its ‘side effect’ lead investigators to focus on safety rather than benefit in patients with cardiovascular disease. PDE5is are again being evaluated in MI and ischaemia–reperfusion.^11 45^ When administered preischaemia and postischaemia PDE5is reduce infarct size, apoptosis, fibrosis and hypertrophy, and postreperfusion increased contractile function and survival^8 11 35^ (figure 4). Mechanisms were PKG-dependent given that both pharmacological and genetic knockdown of PKG abrogate the effects,^46^ while downstream signalling pathways are more contentious, involving mitochondrial function (briefly summarised in figure 5).^11^ Comparative clinical data are lacking, but sildenafil appears to lengthen ischaemia/angina threshold relative to placebo,^47^ and PDE5i users have lower mortality post-MI.^6 7^

Several factors are implicated in the PDE5i cardioprotection in MI.

### Direct myocardial effects

PDE5is activate signalling pathways involved in preventing apoptosis (eg, increased Bcl-2-to-Bax ratio^8^) and improving mitochondrial function. It has been suggested that protective mechanisms occur via postischaemic remodelling given that protection is even observed when PDE5is were administered 3 days postinfarct.^35^

### Coronary artery vasodilatation

Sildenafil in canine ischaemia improved perfusion to myocardial territories,^48^ while in humans with coronary disease without a placebo arm, a single dose of sildenafil vasodilated coronary arteries.^49^ This was accompanied by improved endothelium-dependent vasodilatation, although this was not reproduced in a subsequent double-blinded placebo-controlled crossover study using continuous sildenafil infusion.^50^

### Afterload reduction

PDE5is induce modest transient reductions in BP, while HR is not significantly altered, thus reducing rate-pressure product.^47^

### Antiarrhythmic effects

Studies in animal MI demonstrated antiarrhythmic actions of PDE5is. In ischaemia–reperfusion of isolated rat hearts, sildenafil pretreatment protected against VF accompanied by reduced infarct size and improved LV recovery.^10^ While antiarrhythmic actions could relate to reduced ischaemic burden secondary to coronary artery vasodilatation and afterload reduction, in canine ischaemia, sildenafil pretreatment substantially reduced VAs during coronary occlusion while measures of ischaemia and afterload were unchanged.^12^ PDE5is have electrophysiological effects in cardiac myocytes which may contribute to a direct antiarrhythmic action in ischaemia (figure 2), including reducing *I*_Ca-L_, activating Na^+^/K^+^-ATPase and reducing intracellular Na^+45^, and suppression of β-adrenergic signalling.^22 43^

## Conclusions

Stimulation of the cGMP–PKG axis by PDE5 inhibition provides a novel strategy in the treatment of both HFrEF and MI. While the overall picture is compelling in demonstrating cardioprotection in a drug class with proven safety profiles and widespread use, it is clear that PDE5is act via multiple mechanisms and their efficacy differs according to the pathological process under study. This is depicted in their failure to demonstrate clinical benefit in patients with HFpEF and likely reflects different pathological processes underlying HF subtypes.

Our conclusions are threefold. First, PDE5is have pleiotropic actions in HFrEF comprising (1) pulmonary vasodilatation in HFrEF-PHT, (2) LV remodelling through direct myocardial actions independent of afterload and (3) improved diastolic function. LV remodelling effects do not appear to be significant in HFpEF, and PDE5i use here may only benefit select patients with reversible pulmonary hypertension and RV systolic impairment. Second, PDE5is have acute negative inotropic effects in naive cardiac myocytes and in vivo which may limit utility in HFpEF. Finally, PDE5is compellingly reduce infarct size and have antiarrhythmic properties which, given the context of markedly decreased mortality in PDE5i users at high cardiovascular risk, requires clinical correlation in randomised trials. We propose this could be progressed further in a blinded, placebo-controlled trial of PDE5is in patients with LV impairment following acute MI, examining LV dilatation and remodelling.
